# Functional movement screen dataset collected with two Azure Kinect depth sensors

**DOI:** 10.1038/s41597-022-01188-7

**Published:** 2022-03-25

**Authors:** Qing-Jun Xing, Yuan-Yuan Shen, Run Cao, Shou-Xin Zong, Shu-Xiang Zhao, Yan-Fei Shen

**Affiliations:** 1 Beijing Sport University, School of Sport Science, Beijing, 100084 China; 2 Beijing Sport University, School of Sport Engineering, Beijing, 100084 China

**Keywords:** Risk factors, Computational biology and bioinformatics

## Abstract

This paper presents a dataset for vision-based autonomous Functional Movement Screen (FMS) collected from 45 human subjects of different ages (18–59 years old) executing the following movements: *deep squat, hurdle step, in-line lunge, shoulder mobility, active straight raise, trunk stability push-up and rotary stability*. Specifically, *shoulder mobility* was performed only once by different subjects, while the other movements were repeated for three episodes each. Each episode was saved as one record and was annotated from 0 to 3 by three FMS experts. The main strength of our database is twofold. One is the multimodal data provided, including color images, depth images, quaternions, 3D human skeleton joints and 2D pixel trajectories of 32 joints. The other is the multiview data collected from the two synchronized Azure Kinect sensors in front of and on the side of the subjects. Finally, our dataset contains a total of 1812 recordings, with 3624 episodes. The size of the dataset is 190 GB. This dataset provides the opportunity for automatic action quality evaluation of FMS.

## Abstract (summary)

**Table:** 

Measurement(s)	3D joints coordinates • depth images
Technology Type(s)	depth sensors
Factor Type(s)	movement performance
Sample Characteristic - Organism	Homo sapiens

## Background & Summary

Action quality evaluation plays an important role in various fields: physical rehabilitation^1–4^, posture correction^5^, sentiment analysis^6,7^, and sports training^8–10^. In most cases, experts, such as doctors, physiotherapists, and coaches, evaluate the individual’s physical state by observing specific limb movements based on their extensive experience. This process is inevitably influenced by their subjective attitude^11^, and experienced experts are scarce in many areas. Therefore, developing an automatic action evaluation system is very meaningful.

Building an automatic action evaluation system is not an easy endeavor and involves choosing the most appropriate one from a wide diversity of movement evaluation systems. Considering that the target population of our experiment is the general population, the basic functional abilities that they need to perform is walking, running, and jumping in their daily life. Based on a literature review and the advice of exercise rehabilitation experts, a functional movement screen (FMS) is the most appropriate functional test and was proposed by Gray Cook in the 1990s^12^. It is a simple, efficient screening tool that predicts the risk of sports injury. FMS can comprehensively evaluate the functional performance of individuals according to physical function evaluation criteria. For FMS testing, seven fundamental movements (i.e., *deep squat*, *hurdle step*, *in-line lunge*, *shoulder mobility*, *active straight raise*, *trunk stability push-up* and *rotary stability*)^13,14^ are used to find the defects and deficiencies of the human body in terms of basic flexibility and stability.

FMS research has attracted wide attention in recent years and provided important theoretical and practical perspectives. The reliability and validity of FMS have been discussed for a long time, and numerous studies have confirmed that FMS is a reliable screening tool for physical functional evaluation^15^. In recent years, many application studies have argued that the higher the FMS score is, the lower the risk of sports injury, and individuals with low scores have a significantly increased risk of sports injury. With the rapid advance of sensing elements and computer technologies, some researchers have begun to focus on the direction of intelligent functional movement screens with the help of automatic action evaluation. The study in 2014 by Whiteside *et al*.^16^ compared FMS scores rated by a certified FMS professional with an inertial-based motion capture system consisting of 17 inertial measurement unit (IMU) full-body sensors. Based on the application of a self-set kinematics threshold for FMS scoring, discrepancies can be observed between automatic FMS scoring and manual scoring by professionals. Ross *et al*.^17^ used a data-driven method based on principal component analysis(PCA) and linear discriminant analysis to classify the performance of 542 athletes in seven dynamic screening movements (i.e., *bird-dog*, *drop-jump*, *T-balance*, *step-down*, *L-lop*, *hop-down*, and *lunge*). The accuracy of the model ranged from 70.66% to 92.91%. However, the data for this study were captured by an optical motion capture system, which is expensive to apply. In 2020, the same team^18^ used machine learning methods to train athletes’ movement data captured by IMU, and their model accuracy reached 75.1% to 84.7%, which is a huge improvement. Similarly, the study in 2020 by Wu *et al*.^19^ is an automatic functional movement screening system with 11 IMU sensors. The automatic scoring accuracy of this system can reach 66% 91% using the full movement feature set. Because the cost of inertial sensors is expensive and the intrusive wearing method is inconvenient, functional movement screening systems based on IMUs are not friendly to home-based or broad applications. An automatic action quality assessment system based on a depth sensor becomes a better solution for people to be evaluated at home. Following the system guidance, the user completes the FMS test in front of the camera, and then they can quickly obtain a report of their physical functional quality.

Based on the above considerations, we present a publicly accessible dataset that collected seven FMS movements from 45 participants with two Azure Kinect depth sensors. The dataset contains RGB images, depth images, and skeleton data. In contrast to prior works, a noninvasive depth camera was selected to capture the movement of FMS in our study. Possible applications of the data obtained are:

- Space-time feature representation of functional movements based on 3D skeleton data.
- Action recognition and evaluation of FMS based on noninvasive depth sensors.
- Development of an automatic functional movement screening system with depth sensors.

## Methods

### Participants

The announcement inviting participants for our study was distributed throughout the campus of Beijing Sport University. In the end, forty-five healthy participants (22 women, 25 men), aged between 18 and 59 years (mean = 28.7, s.d. = 10.76), were recruited for this study. Some basic characteristics of the participants were as follows: mass 46–116 kg (mean = 68.8, s.d. = 13.01), height 158–190 cm (mean = 171.9, s.d. = 7.98), and body mass index (BMI) 17.1–32.82 (mean = 23.14, s.d. = 3.1) (Table 1). All participants reported no known movement disorders or other health problems that could affect their mobility. Before starting the experiments, each subject was comprehensively informed about the procedure, introduced to the experimental instrumentations, and informed of any potential risks. Additionally, we required the participants to sign an informed consent form, in which each of them gave their written consent to participate in the research. The study was carried out according to the principles of the Helsinki Declaration and it was approved by the Institutional Review Board of Beijing Sport University. Data collection was completed between May 2021 and June 2021.

**Table 1:** Demographic information of the subjects.

Subject	Age [years]	Gender [F|M]	Weight [kg]	Height [cm]	BMI [kg/m2]
s01	44	M	73	168	25.86
s02	23	F	51	164	18.96
s03	31	F	59	164	21.94
s05	21	M	69	173	23.05
s08	40	F	58	158	23.23
s09	25	M	83	180	25.62
s10	31	F	61	163	22.96
s11	22	M	72	173	24.06
s12	24	M	75	170	25.95
s13	25	M	86	187	24.59
s14	22	F	54	162	20.58
s15	45	M	67	168	23.74
s16	39	M	75	168	26.57
s20	24	M	63	181	19.23
s21	23	M	76	175	24.82
s22	22	M	87	183	25.98
s23	48	F	65	162	24.77
s24	30	F	60	168	21.26
s25	40	F	75	183	22.40
s26	59	F	70	161	27.01
s27	59	F	63	168	22.32
s29	43	F	58.5	163	22.02
s30	21	M	85	181	25.95
s33	49	F	75	162	28.58
s34	26	F	62	172	20.96
s35	38	M	62	168	21.97
s37	21	F	59	160	23.05
s38	32	F	51	168	18.07
s41	20	M	116	188	32.82
s42	20	F	64	175	20.90
s43	20	M	95	190	26.32
s44	20	F	51	171	17.44
s45	24	M	70	174	23.12
s47	20	F	57	168	20.20
s48	18	M	62	173	20.72
s49	20	F	72	176	23.24
s51	20	M	72	178	22.72
s52	24	M	70	172	23.66
s53	24	M	69	176	22.28
s54	24	M	75	177	23.94
s57	24	M	78	182	23.55
s59	22	F	58	164	21.56
s60	20	M	58	177	18.51
s61	22	F	46	164	17.10
s62	23	M	88	178	27.77

All participants’ demographic and anthropometric measurements were carried out during the first session (Table 1).

### Functional movement screen (FMS)

**Fig. 1:**
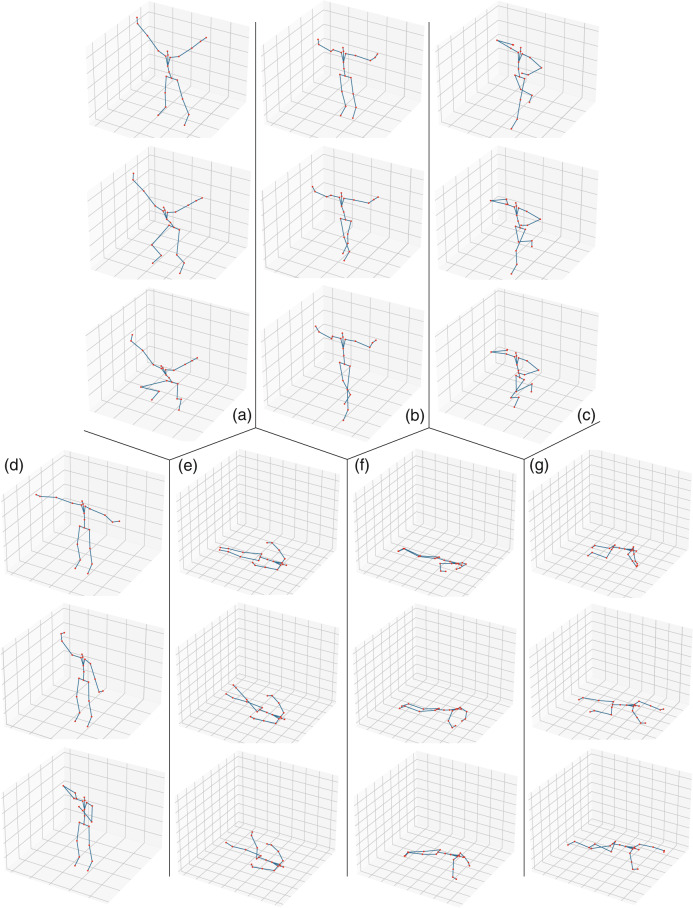
The seven movements of FMS. (**a**) *Deep squat*. (**b**) *Hurdle step*. (**c**) *In-line lunge*. (**d**) *Shoulder mobility*. (**e**) *Active straight raise*. (**f**) *Trunk stability push-up*. (**g**) *Rotary stability*.

FMS is a common screening system that can assess the fundamental movement patterns of an individual^12–14,20^ in sports rehabilitation. It comprises seven fundamental movement tests (Fig. 1, Table 2), which require a balance of mobility and stability, including neuromuscular and motor control^13^. The tests put the individual in extreme positions where weaknesses and imbalance of body become obvious if it is lacking appropriate stability and mobility.

**Table 2:** A brief description of the FMS movements.

Order	Movement	Purpose	Representation in dataset	
			ID	Interpretation
a	Deep squat	Assess bilateral, symmetrical, functional mobility of lower limbs, the shoulders and the thoracic spine, as well as motor control of the core musculature.	m01	Heels on the floor
			m02	Heels on a 2-inch board
b	Hurdle step	Assess bilateral functional mobility and stability of lower limbs.	m03	Left leg up
			m04	Right leg up
c	In-line lunge	Assess mobility, stability, and flexibility of lower limbs.	m05	Left leg in front
			m06	Right leg in front
d	Shoulder mobility	Assess bilateral and reciprocal shoulder range of motion, as well as the scapular mobility and thoracic spine extension.	m07	Left arm up
			m08	Right arm up
e	Active straight raise	Assess hamstring and soleus muscle flexibility while maintaining a stable pelvis and core.	m09	Left leg up
			m10	Right leg up
f	Trunk stability push-up	Assess the ability to stabilize the core and spine, and trunk stability in the sagittal plane.	m11	Support on the ground with both hand
g	Rotary stability	Assess multi-planar trunk stability during a combined upper and lower extremity motion.	m12	Left limb up
			m13	Right limb up
			m14	Left arm and right leg up
			m15	Right arm and left leg up

The FMS movements are broken into two groups–preliminary movement tests (d-g in Table 2, d-g in Fig. 1) and advanced movement tests (a-c in Table 2, a-c in Fig. 1). Each test has an expert score that ranges from 0 to 3 (Table 3). The scoring criteria of FMS are basic, effective, and reliable^21,22^. For more details please refer to these previous studies^13,14^.

**Table 3:** Scoring criteria description of the FMS.

Expert score	Description
3	Complete without compensation
2	Complete with compensation or deviation from the stand, or both
1	Incomplete
0	Complete with pain in any part of the body

### Instrumentation

#### Azure kinect camera

The Azure Kinect sensor^23^ consists of a 12-megapixel (MP) RGB video camera, and a 1-MP depth sensor. The depth sensor’s principle is the time-of-flight (ToF) with amplitude modulated continuous wave (AMCW). The sensor casts modulated illumination in the near-IR (NIR) spectrum onto the scene, and the propagation time for light to travel from the camera to objects in the scene and back to the camera again is measured. These measurements are then processed to generate a depth map, which is a set of Z-coordinate values for every pixel of the image, measured in millimeters^24^. In this way, capturing human body joint points in three-dimensional space by the sensor is made possible.

**Fig. 2:**
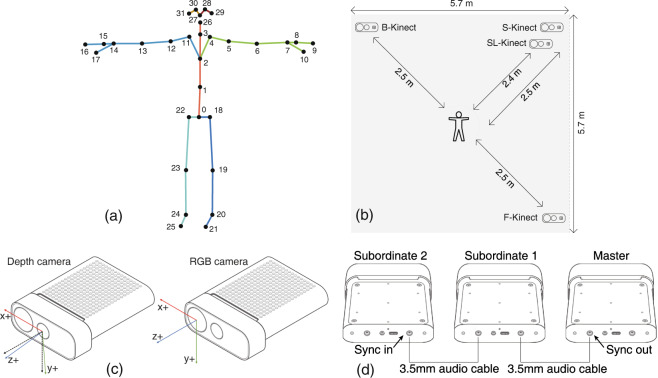
(**a**) Thirty-two joints detected by the Azure Kinect depth sensor. (**b**) Top view of the experimental field. (**c**) RGB and Depth camera coordinate systems^32^. The depth camera is tilted 6° downward of the RGB camera, and the skeleton data are based on this coordinate system. (**d**) Daisy-chain configuration of the multidevice in our experiment^25^.

Microsoft released an Azure Kinect development kit, including a range of software development kits (SDKs) and application programming interfaces (APIs). In our experiments, we used the kit for access to RGB and depth streams and the body tracking SDK, which can track the movements of multiple people simultaneously and provide the three-dimensional coordinates of 32 joints (Fig. 2a, Table 4) per person.

**Table 4:** Joints index and joints names.

Joint index	Joint name	Joint index	Joint name
0	Pelvis	16	Right hand tip
1	Spine navel	17	Right thumb
2	Spine chest	18	Left hip
3	Neck	19	Left knee
4	Left clavicle	20	Left ankle
5	Left shoulder	21	Left foot
6	Left elbow	22	Right hip
7	Left wrist	23	Right knee
8	Left hand	24	Right ankle
9	Left hand tip	25	Right foot
10	Left thumb	26	Head
11	Right clavicle	27	Nose
12	Right shoulder	28	Left eye
13	Right elbow	29	Left ear
14	Right wrist	30	Right eye
15	Right hand	31	Right ear

The data were collected using two Azure Kinect cameras positioned at a height of 96 cm from the ground (F-Kinect and S-Kinect in Fig. 2b). Additionally, FMS experts suggested adding two cameras with different angles so that they could observe the performance of subjects’ movements from multiple angles, improving the accuracy of scoring. Therefore, we also used two auxiliary Azure Kinect cameras positioned at heights of 24 cm and 96 cm from the ground (SL-Kinect and B-Kinect in Fig. 2b). However, we did not acquire the depth image data from these two cameras, and only color images were recorded and published as supplementary data.

All Kinect sensors had the same parameter settings (Table 5). Each Azure Kinect sensor device included 3.5-mm synchronization portals (“Sync In” and “Sync out”), which were used for image synchronization among the different devices. The four sensors were linked together by a 3.5-mm audio cable with a daisy-chain configuration, as shown in Fig. 2d. F-Kinect was the master, S-Kinect was subordinate 1, SL-Kinect was subordinate 2, and B-Kinect was subordinate 3. The four devices’ synchronization adhered to the official documentation instructions^25^. To control the precise timing of each device, the exposure time was first set as 16670 *μ*s by manual exposure, and then an offset of 160 *μ*s was used among the different depth sensor captures to prevent the lasers from interfering with one another.

**Table 5:** Azure Kinect sensor’s config setting.

Camera config	Config setting
RGB camera	1920∗1080 px @30 fps
Color image type	MJPG
Depth camera	640∗576 px @30 fps
Depth image type	DEPTH 16
Internal synchronization	160 *μ*s
External synchronization	0 *μ*s
Exposure time	16670 *μ*s
Field of view (FOV)	75°∗65°(narrow FOV)

#### Computer configuration

To deploy the Azure Kinect DK successfully, the computer configuration needs to satisfy certain prerequisites. Our host operating system is Windows 10 April 2018 (Version 1803, OS Build 17134) release (x64). The hardware system is configured as follows:

- Intel® Core^TM ^i7-7700 CPU @ 3.60 GHZ
- 16 GB Memory
- NVIDIA GeForce GTX 1080
- USB3 port

### Experimental field

As shown in Fig. 2b, we set up a square working scene, surrounded by curtains (6 × 3 m) from three directions, and one side was open to allow the entrance of subjects. While conducting the movements for 01 to 03, the subjects faced F-Kinect, and otherwise, the subjects faced S-Kinect.

### Acquisition protocol

During the entire experiment, the participants visited the laboratory only once. After the participants came to the laboratory, they first watched the video tutorials of FMS, and then a standardized individual warm-up was performed. The warm-up was approximately 3 minutes in duration, which predominantly consisted of stretching exercises. Finally, the participants executed the seven movements of FMS in sequence in the middle of the field (Fig. 2d).

To reduce redundant movement and ensure the purity of the movement sequence, the participants were required to conduct a hold position before starting and to keep this position at the end. The hold position was the state of preparation for each functional movement, which is illustrated by the topmost picture of each part in Fig. 1.

In each movement, the participants were first provided with a short explanation and movement demonstrations by an experimenter. Then, they were instructed to perform each movement of FMS in turn in the most natural way, and every movement was repeated three times except for shoulder mobility. Each subject spent approximately 50–60 min finishing the process of data acquisition. The total time to obtain all of the experimental data was approximately 5 days.

Not all movements were performed by all participants. For example, a few participants had to stop challenging movements because of physical pain, and their scores for the corresponding movements were labeled zero, which indicated that their bodies had functional defects.

In Table 2, the rightmost column provides an explanation of the movement number in our dataset and the corresponding relationship with the seven movements of FMS. For instance, the *deep squat* of FMS includes two situations, i.e., m01 and m02, where m01 is whole sole touching the floor, and m02 is the heels on the block. The former is more difficult than the latter. These two movement patterns make the functional evaluation of participants easier.

## Data Records

Our own data acquisition software, based on official documents^26^ and APIs of Azure Kinect, was used to obtain the data (MKV format) from the sensors. All raw data were stored as videos (MKV format) within hard disk drives. The storage of all video files reached 7.3 Tb. After preprocessing frame extraction and skeleton extraction, the image files and skeleton data files were obtained and published as our dataset. The dataset mainly contained color images (JPG format), depth images (PNG format), human body skeleton data (JSON format), and expert scoring (JSON format). We anonymized each image by detecting faces and then blurring them to ensure that the subjects’ privacy was protected and that no face could be recognized. The dataset is available at Figshare (10.25452/figshare.plus.c.5774969)^27^. Please refer to the Code Availability for the related codes.

### Dataset organization

**Fig. 3:**
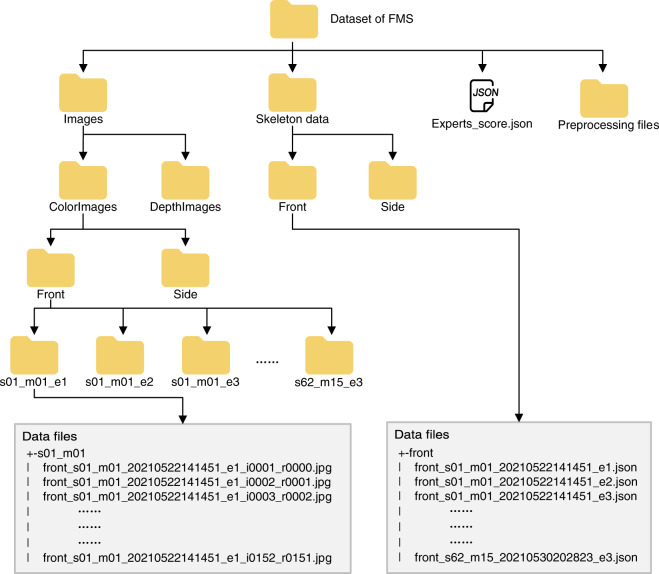
Dataset’s directory structure.

In the dataset, all data are contained in the *FMS* folder. The directory structure is shown in Fig. 3. Each directory contains several subfolders corresponding to the movements recorded. The content of the subfolders are introduced in the following:

- **Images**: the folder containing all color images and depth image files.
- **ColorImages**: the folder containing all color image files.
- **DepthImages**: the folder containing all depth image files.
- **Front**: the folder containing data from F-Kinect.
- **Side**: the folder containing data from S-Kinect.
- **s01_m01_e1**: the folder containing images for the 1st episode of the 1st movement performed by the 1st subject. The other directories are the same.
- **Skeleton data**: the folder containing all skeleton data files (JSON format).
- **Experts_score.json**: the file containing the three FMS experts’ scores for all episodes of movements.
- **Preprocessing files**: the folder containing files required or generated during the process of data preprocessing.

The dataset includes 3624 skeleton JSON files, of which 1812 files are from the front position and 1812 files are from the side position. Simultaneously, there are 3624 sets of color images and 3624 sets of depth images in the dataset. Each set corresponds to one episode of a movement. The JSON files and image files are created in the format of *SensorPos_subjectID_movementID_timestamp_episodeNo.json* and *SensorPos_subjectID_movementID_timestamp_episodeNo_episode frameID_movement frameID.jpg/png*, respectively, and the detailed explainations about the nomenclature are as follows:

- **SensorPos**: the position of the Azure Kinect sensor relative to the subjects, i.e., ‘front’ or ‘side’.
- **subjectID**: index of subjects, e.g., s01, s02, etc.
- **movementID**: index of movements, e.g., m01, m02, etc.
- **timestamp**: the beginning time of movements, in the format “YYYYMMDDHHMMSS”.
- **episodeNo**: index of the episodes of movements, e.g., e1, e2, e3.
- **episode frameID**: index of episode frames, e.g., i0001, i0002, i0003.
- **movement frameID**: index of movement frames, e.g., r0001, r0002, r0003.

For example, the skeleton data file *front_s01_m02_20210522141451_e3.json* refers to the 3rd episode of the 2nd movement performed by the 1st participant, at 14:14:51 on May 22, 2021. Different from the JSON files, the nomenclature of the images files also include information about the frame index. For example, *front_s01_m04_20210522141847_e2_i0001_r0213.jpg/png* refers to the 1st frame of the 2nd episode, which is the 4th movement performed by the 1st participant, at 14:18:47 on May 22, 2021. Meanwhile, this frame is the 213th frame of the 4th movement.

In our dataset, the skeleton data and expert score data are stored in JSON format, which uses human-readable and machine-processable text to store the transmit data objects consisting of collections of attribute-value pairs and ordered lists of values. The internal structure of the JSON files is explained in the following section.

#### Internal structure of the skeleton data files

Each episode of movements has a JSON format document, which stores skeleton data. Box 1 shows a document of the 1st episode of the 1st movement performed by the 1st participant. It contains information as follows:

- **frames**: a list of skeleton data from all frames of the episode in frame order.
- **original_frame_num**: original frame number in the movement for each frame in episode.
- **total_frames**: frame number of the episode.
- **bodies**: a list of skeleton data for all tracked people. It contains everyone’s skeleton data in the frame (in our dataset, there is only one person per frame.)
- **frame_id**: the index of the current frame.
- **num_bodies**: the number of people in the frame.
- **timestamp_usec**: the timestamp of the current frame.
- **body_id**: the index of the tracked body in the current frame.
- **confidence_level**: a list of the confidence level of the joints (the index of each item corresponds to the index of the joint).
- **joint_orientations**: a list of quaternions of the joint orientation values (in the order of w, x, y, z).
- **joint_position_2d_color**: a list of 2D pixel coordinates (on the order of x, y) of the joints in the color image.
- **joint_positions**: a list of Cartesian coordinates of the joints in the camera coordinate system.

Box 1 Information of the document front_s01_m01_20210522141451_e1.json {“frames”:[{“bodies”:[{“body_id”: 1,“confidence_level”: [2, 2, 2,...],“joint_orientations”:[[0.5473520159721375,

−0.4423481523990631,

 0.5430209636688232,

−0.4581070840358734],...],

“joint_position_2d_color”:[[985.2445068359375,577.977294921875],...],“joint_positions”:[[129.94537353515625,

−243.37063598632812,

3150.84033203125],...]}],“frame_id”: 0,“num_bodies”: 1,“timestamp_usec”: 156033},...],“original_frmae_num”: [0, 1, 2,...],“total_frames”: 152}

#### Internal structure of expert score files

In our dataset, each episode of movements was annotated by three FMS experts, and the annotation files were stored in the document of *experts_ score.json*. Box 2 shows the internal structure of the document, which is a nested relationship. It contains information as follows.

- **s01/s02/s03/**: the keys are the subject IDs and the subfield of the key stores expert scores of all movements by the corresponding subject.
- **m01/m02/m03/**: the keys are the movement IDs and the subfield of the key stores expert scores of all episodes of the corresponding movement.
- **e1/e2/e3**: The keys are the episode IDs, and the subfield is a list that stores the expert scores of the corresponding episode.

Box 2 Information of the document score_by_three_experts.json {“s01”: {“m01”: {“e1”:[2, 2, 2],“e2”:[2, 2, 2],“e3”:[2, 2, 2]},“m02”: {...},“m03”: {...},...},“s02”: {...},“s03”: {...},...}

## Technical Validation

### Sensor accuracy

The technical validation of the Azure Kinect sensor has been reported in previous studies^28–30^, which indicates that the officially stated values are accurate, namely s.d. ≤17 mm, and distance error <11 mm. Hence, we do not provide test-retest reliability information about the sensor.

### Comparison with published datasets

We have not found a public dataset of FMS on the major journal websites of sports sciences. Although a few researchers have begun to study the automatic evaluation of FMS with the help of wearable inertial sensors, their datasets have not been publicly available. In addition, the tester has to wear cumbersome inertial sensors that cannot be operated by individuals at home. To tackle these problems, we propose a vision-based FMS dataset that provides help for the process of transition from marker-based methods to marker-less methods.

### Missing episodes and frames

Limited by the physical condition of some subjects and the difficulty of *rotary stability*, 37 episodes were not well executed as planned: 11 episodes for m12, 19 episodes for m13, 5 episodes for m14 and 2 episodes for m15. These missing episodes were recorded in the *missing movement episodes.xlsx* file (in *Preprocessing files* folder). This phenomenon also showed that *rotary stability* seems to be the most difficult movement in FMS.

**Fig. 4:**
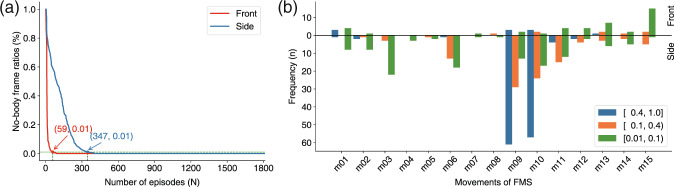
No-body skeleton data frame distribution of the front and side Azure Kinect sensors. (**a**) Number of episodes with no-body frames captured by the front and side sensors. (**b**) Distribution of episodes with a no-body skeleton frame in the movements.

Because the depth sensor can barely recognize the human skeleton under severe self-occlusion situations, skeleton data of such frames are missing and this information is stored in the *Statistics of frames without skeleton* folder. For these missing skeleton data, interpolation can be performed. Overall, the number of episodes containing no-body skeleton frames recorded by the front sensor is less than that from the side sensor (Fig. 4a). Given the frame number of missing data *N*_*nobody*_ and the total frame number *N*_*total*_ in one episode, we can calculate the loss rate (*γ*) of skeleton data as follows:

1 $$\gamma =\frac{{N}_{nobody}}{{N}_{total}}\times 100 \% .$$

Episodes with no-body skeleton frames in the statistical analysis met the condition of *γ* ≥ 0.01. There were 59 episodes with no-body skeleton frames recorded by the front Kinect and 347 episodes with no-body skeleton frames by the side Kinect in our dataset (Fig. 4a). This shows that the problem of self-occlusion is more serious for the side sensor, and the angular position relationship between the human body and camera should be set carefully while capturing human skeleton data with depth cameras. In Fig. 4b,we notice that the *active straight raise* (m09, m10) is the movement that contains the most no-body skeleton episodes, which indicates that the position between this movement and the camera should be adjusted appropriately to improve the accuracy of the action quality evaluation results.

### Sensor synchronization

We synchronized the front and side Azure Kinect sensors according to the instructions of the official document^25^, and the color images of the two sensors were completely synchronized. To prevent the lasers from interfering with one another, the front sensor was offset from the side sensor by 160 *μ*s or more, so depth images of the two sensors differed by 160 *μ*s in our dataset. In the case of synchronization, the frame number of the same episode captured by the front and side sensors should be the same.

**Fig. 5:**
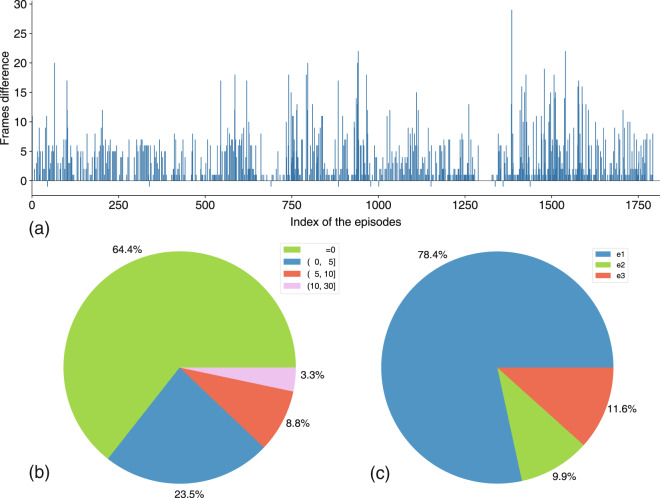
Distribution of missing frames. (**a**) Frame number difference of all episodes captured by the front and side sensors. (**b**) Distribution of frames number difference. (**c**) Distribution of frame loss in different episodes.

However, frame loss is always inevitable. To obtain an overall view of this problem, we report some statistical results of frame loss in Fig. 5. The difference of frame number for the same episode (total 1812 episodes) captured by two sensors was always less than 30 (Fig. 5a), and the frame number of 64.4% (1167) episodes captured by the two sensors was the same. Specifically, the frame number difference for 23.5% (426) of the episodes captured by the two sensors was less than 5, the difference in the frame number for 8.8% (159) of episodes captured by the two sensors was less than 10, and only 3.3% (60) of the episodes captured by the two sensors had a frame difference greater than 10 (Fig. 5b). Notably, 78.4% of episodes that lose frames belonged to the 1st episode (Fig. 5c), and most of them happened in the first 60 frames (1st & 2nd second, 30 fps) of the episodes. This also indicates that the workflow is unstable for just a few seconds after the camera is turned on, and the sensor needs to be warmed up for a time. For the image frames lost in the segment, the video frame interpolation method can be used to compensate.

### Distribution of expert scoring

To provide a new dataset for the automatic action evaluation system of FMS, three FMS experts with rich experience (at least two years) completed the FMS screening for each subject. The experts included a teacher of Sport Medicine and Physical Therapy Department of Beijing Sport University with a PhD in Sport Science (expert 1), a researcher of Chinese Academy of Sport and Health of Beijing Sport University with a PhD in Sport Science (expert 2), and a teacher of Strength and Conditioning Department of Beijing Sport University (expert 3). The movement performance of the subjects was rated by watching the videos from four angles simultaneously with experts according to the standard FMS scoring rules^13,14^. The interrater reliability of the FMS experts resulted in an ICC of 0.928 (95% CI:0.878, 0.958) and was considered good. The FMS composite scores ranged from 8 to 18 points (mean = 13.1, median = 13 points, s.d. = 2.18).

**Fig. 6:**
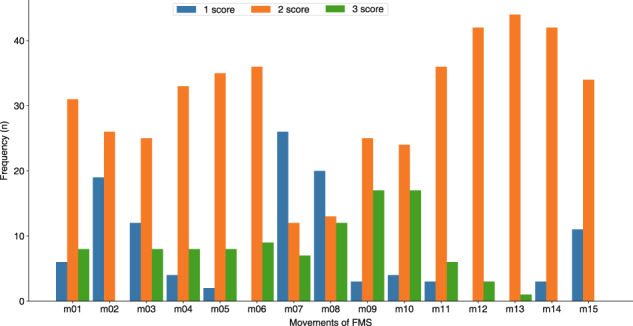
Expert score distribution of FMS movements.

The score distribution of each movement is shown in Fig. 6. Because the number of executions for the same movements performed by different subjects may vary, the expert score *S*_*m*_ of one movement by a certain subject is a statistical sample, which was calculated as follows:

2 $${S}_{m}=round\left(\frac{1}{KN}\mathop{\sum }\limits_{i=1}^{K}\mathop{\sum }\limits_{j=1}^{N}{S}_{ij}\right),$$

where *round(.)* means the rounding function and *S*_*ij*_ denotes the score of the *i* th episode by the *j* th expert. Each movement has *K* ($K\in \{1,2,3\}$) episodes, and each episode is scored by *N* (*N* = 3) experts.

From Fig. 6, we observe that the score distribution of each movement is approximately Gaussian. Movements with a score of “2” are the most common, followed by a score of “1”, and finally a score of “3”. Because of the difficulty in conducting the movement of *rotary stability*, this movement has almost no scores of “3”. Moreover, Shoulder mobility (m07, m08) is the movement that obtained the highest score of “1”, which reflects that shoulder injuries are common.

### Movement features

Spatiotemporal features are very important for action recognition and evaluation. To automatically evaluate the movements of FMS, the 3D coordinates and angles of joints can be used. For each movement, if the peak values of the coordinates and the angles exceed a specified threshold, movement has occurred. Moreover, the temporal information of the coordinates and angles can reflect the quality of movement. For the movements of FMS, we analyzed the trajectories of the coordinates and angles of the major joints.

The direction of the vertical axis of the camera coordinate system is from top to bottom (Fig. 2c). To be consistent with the actual directions of human movement, we reverse the vertical axis of the camera coordinate system (i.e., reflection transformation) to create a new y-axis in our analysis.

**Fig. 7:**
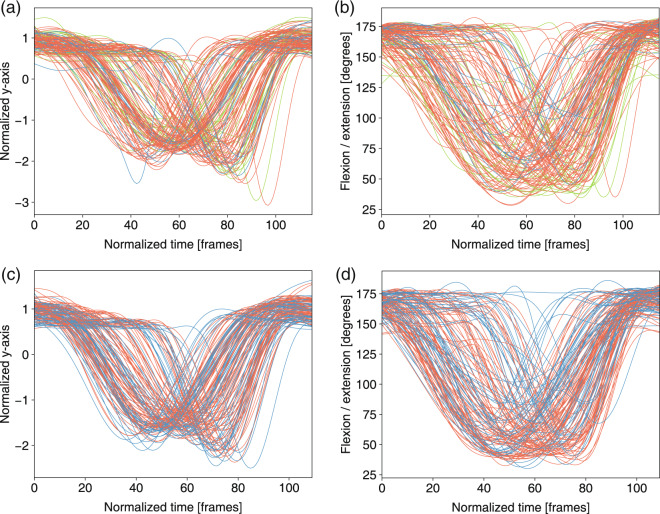
Left hip joint trajectory and left knee angles in *deep squat* for three levels: 3 score (green), 2 score (orange), 1 score (blue). (**a**) Normalized y-axis of trajectory of the hip joint in m01. (**b**) Flexion/extension knee angle in m01. (**c**) Normalized y-axis of trajectory of the hip joint in m02. (**d**) Flexion/extension knee angle in m02.

**Fig. 8:**
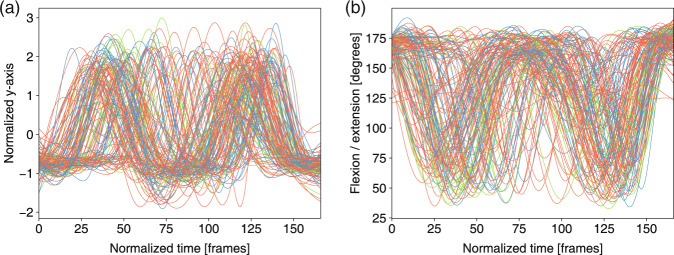
Left knee joint trajectory and angles in *hurdle step* for three levels: 3 score (green), 2 score (orange) and 1 score (blue). (**a**) Normalized y-axis of the trajectory of the knee joint in m03. (**b**) Flexion/extension left knee angle of the leg in m03.

**Fig. 9:**
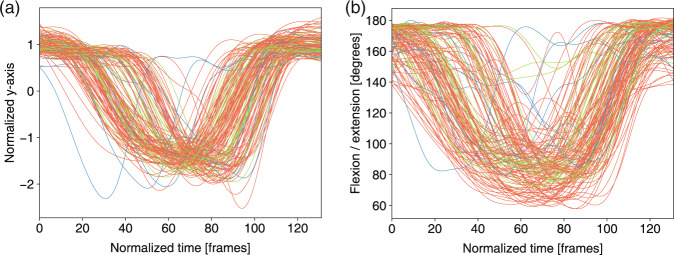
Left hip joint trajectory and left knee angles in *in-line lunge* for three levels: 3 score (green), 2 score (orange), 1 score (blue). (**a**) Normalized y-axis of trajectory of the hip joint in m05. (**b**) Flexion/extension knee angle of the left leg in m05.

**Fig. 10:**
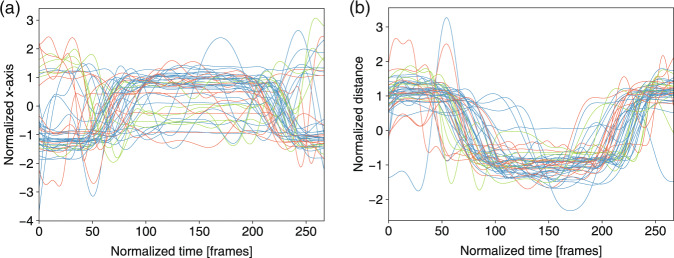
Left wrist joint trajectory and wrist spacing in *shoulder mobility* for three levels: 3 score (green), 2 score (orange), 1 score (blue). (**a**) Normalized x-axis of trajectory of the wrist joint in m07. (**b**) Normalized wrist spacing in m07.

**Fig. 11:**
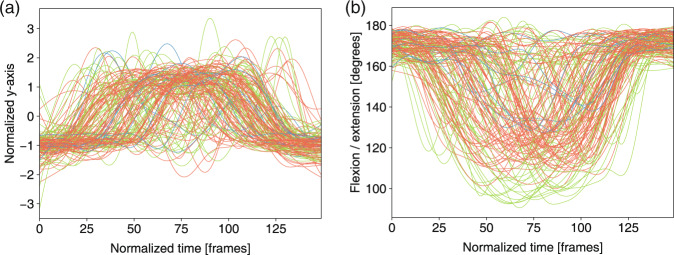
Left ankle joint trajectory and the angle ranges between the horizontal plane and the test leg in *active straight raise* for three levels: 3 score (green), 2 score (orange), 1 score (blue). (**a**) Normalized y-axis of the trajectory of the ankle joint in m09. (**b**) Angle ranges between the horizontal plane and the test leg in m09.

**Fig. 12:**
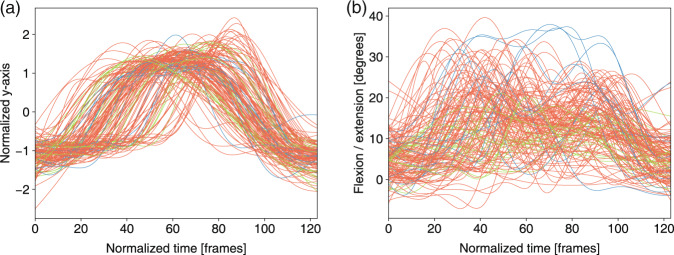
Neck joint trajectory and the angle ranges between the horizontal plane and thoracic vertebra in *trunk stability push-up* for three levels: 3 score (green), 2 score (orange) and 1 score(blue). (**a**) Normalized y-axis of the trajectory of the neck joint in m11. (**b**) Angle ranges between the horizontal plane and the thoracic vertebra in m11.

**Fig. 13:**
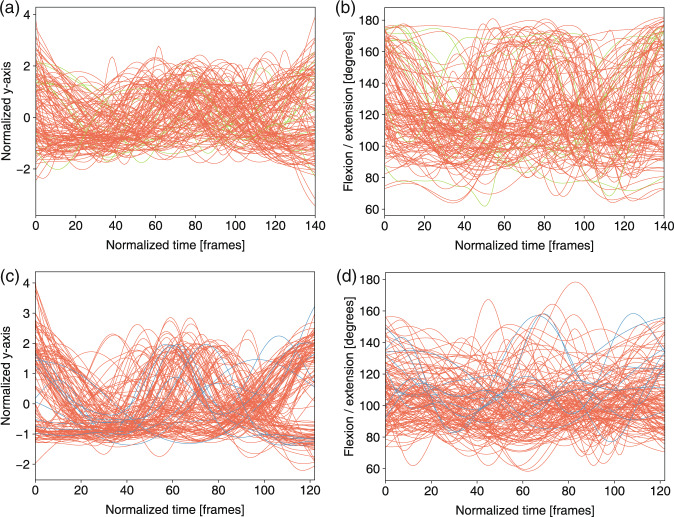
Knee joint trajectory and angles in *rotary stability* for three levels: 3 score (green), 2 score (orange) and 1 score (blue). (**a**) Normalized y-axis of the trajectory of the left knee joint in m12. (**b**) Flexion/extension of the left knee angle in m12. (**c**) Normalized y-axis of the trajectory of the left knee joint in m14. (**d**) Flexion/extension knee angle in m14.

To perform a trajectory analysis of the joint features (i.e., the joint coordinates and joint angles), we applied a three-step normalization method in this part. A Butterworth filter was used to remove the noise as the first step. The second step was to interpolate the feature data into a predefined length. The last step was to normalize the data using a z-score of *d* (the time series of coordinates x, y, and z):

3 $$Z=\frac{(d-\mu )}{\sigma },$$

where *μ* and *σ* are the mean value and the s.d. of *d*, respectively. In Figs. 7–13, for the analysis of the joint angles, only the first two steps were used, and all three steps were used for the joint trajectory analysis.

The feature trajectories over time for all of the movements are shown in Figs. 7–13, and we visualized the trajectories of the joints that had clear change in value. Each score was drawn using a different color: (i) the score of “1” (blue), (ii) the score of “2” (orange) and (iii) the score of “3” (green). The feature trajectories of the same movement are similar. Every movement is presented from the preparatory phase to the intermediate phase and then to the final phase.

For the *deep squat* (Fig. 7), the hip trajectory is presented on the y-axis (Fig. 7a,c), and the knee angle is the basic feature of *deep squat*, with a large range from 180° to 40° in Fig. 7b,d. Moreover, the comparison between Fig. 7a–d shows that m02 is a simplified version of m01, and its maximum score is 2. For the *hurdle step* (Fig. 8), the knee trajectory is presented on the y-axis (Fig. 8a) and the angle range of the knee joint is the same as that on the *deep squat*, but with one more round trip phase. For the *in-line lunge* (Fig. 9), the hip trajectory is presented on the y-axis (Fig. 9a), and the angle range of the knee joint is from 180° to 60° (Fig. 9b). The trajectory of the “1” score is more unstable. Compared with the other movements, *shoulder mobility* (Fig. 10) requires the subjects to maintain the same position for a long time (250 normalized times) and perform the movement only once. For the *active straight raise* (Fig. 11), Fig. 11a shows the ankle trajectory on the y-axis, and Fig. 11b shows that the angle range between the horizontal plane and test leg ranges from 100° to 90°. The round trip phase is significantly more abundant than the normal phase, which is caused by the instability of the human body recognition algorithm in cases of self-occlusion. For the *trunk stability push-up* (Fig. 12), the neck trajectory is presented phase y-axis (Fig. 12a), and Fig. 12b shows the changes in the angle between the horizontal plane and thoracic vertebra. The angle variation range of the “1” score is larger, while that of the “3” score is the smallest. For *rotary stability* (Fig. 13), the ankle x-axis trajectory is presented in Fig. 13a,c, and the knee angle range is presented in Fig. 13b,d. The messy curves in the figure show that most subjects cannot execute the movement correctly, and the *rotary stability* is the most difficult movement in the FMS.

### Joint position and orientations

In this section, we analyzed the range of motion of the lower limb joints (left hip, left knee, left ankle) in *deep squat* and the minimum wrist spacing in *shoulder mobility*. All data were from the front Azure Kinect sensor and low pass filtered at 50 Hz using a second-order Butterworth filter.

The mean joint angle range in the *deep squat* was first recorded with the Azure Kinect body tracking SDK and then compared to the results of 3D Vicon motion analysis system^31^ as shown in Table 6. Angles obtained via the 3D Vicon motion analysis system were 24.1° for ankle dorsiflexion, 109.9° for hip flexion and 87.3° for hip flexion. Measurements derived with the Azure Kinect body tracking SDK were 32.0°, 114.1°, and 89.2° for the ankle, knee and hip, respectively. From Table 6, we can observe that differences between the two vision-based systems at the hip, knee and ankle were slight under different experimental conditions, with mean differences of 7.9°, 4.2° and 1.9°, respectively. This phenomenon shows that the values of the joint position and orientations obtained from the Azure Kinect body tracking SDK are reasonable. Moreover, the differences may be due to various factors, such as the different subjects and the measurement system.

**Table 6:** Comparison of the joint angle range of *deep squats* in our experiments with the results of the 3D Vicon motion analysis system.

Variable	Our Study	David Krause *et al*.^31^
	Mean ± S.d.	Mean ± S.d.
Ankle dorsiflexion Range (deg)	32.0 ± 8.4	24.1 ± 6.6
Knee Flexion Range (deg)	114.1 ± 17.8	109.9 ± 17.6
Hip Flexion Range (deg)	89.2 ± 17.3	87.3 ± 10.5

Additionally, the range of motion of the ankle, knee and hip in *deep squat* as well as the minimum wrist spacing in *shoulder mobility* were analyzed to examine differences in the performance among these types of FMS scoring. These data were statistically analyzed using the Kruskal-Wallis test, performed using SPSS v.25 (SPSS, Chicago, IL, USA). Statistical significance was identified at *p* < 0.05 except for the range of motion of the ankle (*p* = 0.427) (see Table 7). This suggests that the range of changes in the hip and knee angles can better distinguish the quality of performance in the *deep squat* than the range of ankle flexion angles. In *shoulder mobility*, the minimum wrist spacing can better distinguish the quality of performance. Based on the above results, we believe that the range of joint angles can be used as a feature to improve the accuracy of scoring in the vision-based automatic evaluation model. Meanwhile, more movement features should be added to further improve the accuracy of the model prediction.

**Table 7:** Joint angle excursion during the *deep squat* and wrist spacing in the *shoulder mobility*.

Variable	Score 3		Score 2		Score 1		*p*
	Mean ± S.d.	Median	Mean ± S.d.	Median	Mean ± S.d.	Median	
Ankle Dorsiflexion Range (deg)	31.6 ± 5.4	29.7	32.9 ± 7.5	34.7	31.3 ± 10.1	29.7	0.427
Knee Flexion Range (deg)	117.5 ± 17.6	124.2	121.1 ± 13.2	119.2	103.9 ± 22.9	106.9	0.000
Hip Flexion Range (deg)	97.1 ± 19.4	100.2	94.4 ± 6.9	93.5	80.0 ± 20.9	84.1	0.000
Wrists spacing (cm)	29.9 ± 9.8	29.8	32.9 ± 7.8	31.7	40.3 ± 9.3	41.9	0.001

*p* < 0.05 indicates significant differences among the three grades.

### A baseline analysis

In this section, we provide a baseline analysis for the automatic FMS movement assessment tasks. Here, only data from the front view camera are processed, and 3D skeleton sequences extracted from the original videos are used as input directly. Training and testing data are performed on the same view. As the most common metrics used for classification in machine learning, the F1 score and accuracy are reported in our study.

**Fig. 14:**
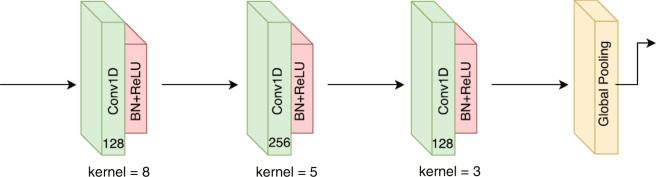
The structure of FCN network^33^.

Generally, our baseline model has three major phases: data preprocessing, feature representation, and classifier model. The estimated skeleton data can often be noisy in real scenes. To obtain robust feature data, normalization and alignment are performed first to deal with intraclass variations. Second, a simple network composed of a fully convolutional network (FCN) architecture (refer to Fig. 14 for the network’s structure) and one fully connected layer is adopted to learn effective feature representation from 3D skeleton joints. Finally, the FMS scoring (1–3 points) is predicted with the help of a softmax classifier. Table 8 shows the FMS movement assessment results for the testing data, presented for each folder of the 5-fold cross-validation. The automatic evaluation performance for different FMS movements with vision-based sensors is reported separately.

**Table 8:** Performance of the action quality assessment for seven different FMS movements.

Movement	F1 score					Accuracy (%)				
	Folder1	Folder2	Folder3	Folder4	Folder5	Folder1	Folder2	Folder3	Folder4	Folder5
Deep squat	0.728	0.761	0.770	0.736	0.534	80.8	84.7	84.6	85.2	66.0
Hurdle step	0.629	0.610	0.730	0.681	0.622	66.7	75.0	77.6	83.3	72.0
In-line lunge	0.420	0.537	0.677	0.836	0.435	79.2	87.3	83.1	92.6	79.2
Shoulder mobility	0.379	0.367	0.365	0.345	0.196	52.0	46.7	37.5	38.1	38.5
Active straight raise	0.725	0.739	0.657	0.757	0.664	83.3	77.1	70.2	80.7	72.2
Trunk stability push-up	0.713	0.472	0.582	0.881	0.269	80.8	0.848	79.2	95.7	67.7
Rotary stability	0.535	0.675	0.519	0.396	0.321	88.8	86.7	91.9	85.9	92.9
Overall	0.685	0.699	0.702	0.693	0.608	78.2	81.8	80.7	83.4	77.1

## Usage Notes

The dataset is available at Figshare (10.25452/figshare.plus.c.5774969). For more detailed information about Azure Kinect, refer to the Azure Kinect DK documentation website (https://docs.microsoft.com/en-us/azure/Kinect-dk/). For anything not covered, please contact Qing-Jun Xing at xqj_fitness@163.com.

## Data Availability

Our codes for data acquisition and data processing are provided on the following Github repository: https://github.com/sselab2021/FMS_human_3d-skeleton_by_Azure-Kinect.
